# Pharmacokinetics, Bioavailability, and Tissue Distribution Study of Angoroside C and Its Metabolite Ferulic Acid in Rat Using UPLC-MS/MS

**DOI:** 10.3389/fphar.2018.01186

**Published:** 2018-10-23

**Authors:** Chenning Zhang, Weidong Ma, Yonghong Zhang, Qibin Wang, Caibin Qin, Shiming Du, Liangyong Huang, Fang Ye, Li Chen, Tao Zheng

**Affiliations:** ^1^Institute of Wudang Traditional Chinese Medicine, Taihe hospital, Hubei University of Medicine, Shiyan, China; ^2^Department of Pharmacy, Taihe Hospital, School of Medicine, Xi’an Jiaotong University, Shiyan, China; ^3^Hubei Key Laboratory of Wudang Local Chinese Medicine Research, Hubei University of Medicine, Shiyan, China

**Keywords:** angoroside C, ferulic acid, pharmacokinetics, tissue distribution, UPLC-MS/MS

## Abstract

Angoroside C is a phenylpropanoid glycoside compound isolated from the dried root of *Scrophularia ningpoensis* Hemsl., which possesses the effects of preventing ventricular remodeling, reducing pulmonary oedema, and reducing blood pressure, as well as having the properties of anti-platelet aggregation, hepatoprotection and anti-nephritis, etc. However, few investigations have been conducted on the absorption, distribution, metabolism, and excretion (ADME) study of angoroside C. Thus, a fast ultra-high performance liquid chromatography-tandem quadrupole mass spectrometry (UPLC-MS/MS) method was established for the determination of angoroside C and its metabolite ferulic acid in rat plasma and tissue homogenate. The two analytes were extracted from the biosamples using a simple protein precipitation with acetonitrile. The developed method was validated and successfully applied to the pharmacokinetics, bioavailability and tissue distribution study after the intragastric administration of angoroside C (100 mg/kg) or the intravenous administration of angoroside C (5 mg/kg), respectively. The results showed that angoroside C can be absorbed extremely quickly (*T*_max_ = 15 min), can be eliminated very rapidly (*t*_1/2_ = 1.26 h), and its oral bioavailability is only about 2.1%. Furthermore, angoroside C was extensively distributed in all main organs (liver, heart, spleen, lung, kidney, and brain), and the highest concentration was detected in the lung 15 min after oral administration. This paper also indicated that angoroside C could be converted to the active metabolite ferulic acid *in vivo*. The maximum concentrations of ferulic acid in the kidney occurred at 6 h after oral administration. In summary, this study explored some of the pharmacokinetic characteristics of angoroside C *in vivo*, and the data produced could provide a basis for the further investigation of angoroside C.

## Introduction

Radix Scrophulariae is the name for the dried roots of *Scrophularia ningpoensis* Hemsl. (National Pharmacopoeia Committee, 2015), and is a famous botanical medicine used widely in Asian populations. First recorded in “ShennongBencao Jing,” Radix Scrophulariae contains a variety of chemical ingredients, such as iridoid, phenylpropanoid, triterpenoid saponins, etc. (Xu et al., 2013). Modern pharmacological researches have shown that Radix Scrophulariae possesses anti-inflammatory effects, and is able to protect the cardiovascular system, and enhance immunity, among other properties (Qian et al., 1991).

**FIGURE 1:**
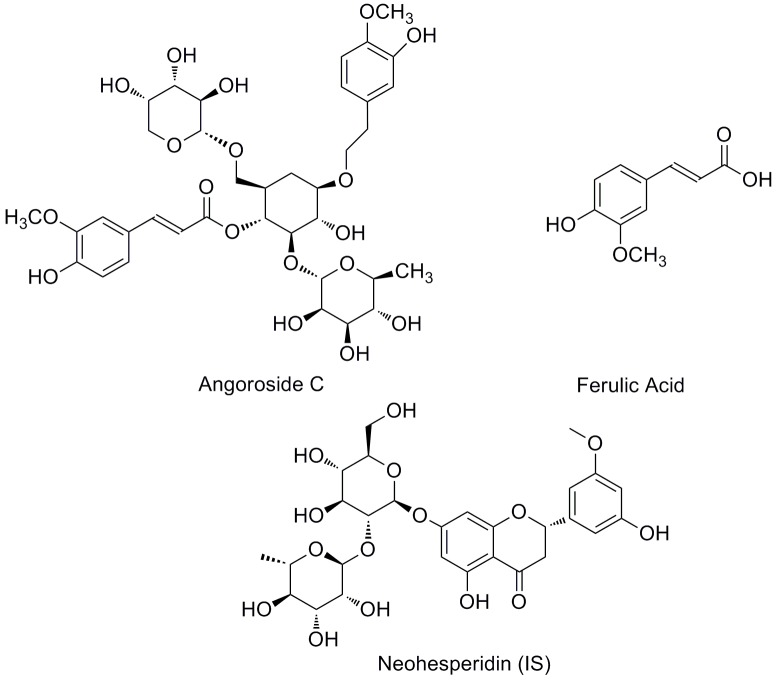
Chemical structures of angoroside C, ferulic acid, and neohesperidin (IS).

Angoroside C (Figure 1) is a representative phenylpropanoid glycoside (PhG) compound isolated from *Scrophularia ningpoensis* Hemsl (Li et al., 2000; Wagner et al., 2011). Which possesses the ability to reduce blood pressure (Liu et al., 2015), and has anti-inflammatory (Díaz et al., 2004), anti-platelet aggregation (Cano et al., 1990), hepatoprotection (Huang et al., 2004; Morikawa et al., 2010), and hypoglycemic properties (Tian et al., 2017), as well as being able to reduce pulmonary oedema (Zhang et al., 2017). Furthermore, according to the continuous series of in-depth studies by Gu Weiliang on Radix Scrophulariae (Gu et al., 2010; Huang et al., 2012), the results showed that angoroside C has beneficial effects against ventricular remodeling, and the mechanism is likely to be related to decreasing the level of Ang II, attenuating the mRNA expressions of endothelin 1 (ET-1) and transforming growth factor β1 (TGF-β1) (Gu et al., 2015), which is effective in postponing heart failure for patients with cardiovascular illness.

In our previous study, we found that angoroside C can be metabolized to ferulic acid in rats, which is one of the functional phenolic acids that commonly exist in a variety of plants, and multiple biological and pharmacological properties such as anti-oxidant (Perezternero et al., 2017), anti-inflammatory (Salazar-López et al., 2017), anti-obesity (Salazar-López et al., 2017), and anti-depression (Liu et al., 2017b) have been reported for this compound. Most important of all, ferulic acid has been demonstrated to have protective effects against myocardial ischaemia (Liu et al., 2016; Panda et al., 2016), which is usually considered to induce ventricular remodeling. Therefore, it is important and meaningful to pay more attention to the pharmacokinetic characteristics of ferulic acid *in vivo* after the oral administration of angoroside C during pharmacodynamic evaluations.

To date, some HPLC methods were applied in determine angoroside C in Radix Scrophulariae (Yang et al., 2009; Zhang et al., 2011), and only one paper reported the LC-MS/MS method to determine multi-components including angoroside C in plasma after intragastric SimiaoYong′an decoction to rat (Liu et al., 2017a). But beyond that, no significant ADME studies have been published in the literature on angoroside C and its metabolites *in vivo*. The purpose of this paper is to develop a sensitive and reproducible UPLC–MS/MS methods to simultaneous quantify angoroside C and its metabolite ferulic acid and apply the method to rat pharmacokinetics, bioavailability, and tissue distribution studies. We hope that the results will provide a basis for elucidating the pharmacodynamic effects of angoroside C and the clinical use of Radix Scrophulariae.

## Materials and Methods

### Chemicals and Reagents

Angoroside C (purity > 98%) and the internal standard neohesperidin (IS; purity > 98%) were purchased from Chengdu Must Bio-Technology Co., Ltd. (Chengdu, China). Ferulic acid (Figure 1) (purity > 98%) was obtained from the National Institutes for Food and Drug Control (Beijing, China). HPLC grade acetonitrile and methanol were purchased from Merck (Darmstadt, Germany). Distilled water was prepared in our lab using a GWA-UN ultra-pure water apparatus (Purkinje General, China). All other chemicals and reagents were of analytical grade and purchased from certified vendors.

### Apparatus and Conditions

Liquid chromatography was performed on an Acquity^TM^ ultra performance liquid chromatography (UPLC) unit (Waters Corp., Milford, MA, United States) with a Waters HSS T3 column (50 mm × 2.1 mm, 1.8 μm) (Waters Corp., Milford, MA, United States). A gradient elution program was conducted for chromatographic separation, with the mobile phase A (0.1% formic acid–water) and mobile phase B (0.1% formic acid–acetonitrile) as follows: 0–0.5 min (5–5% B), 0.5–4.5 min (5–95% B), 4.5–5.0 min (95–95% B), 5.0–6.5 min (95–5% B), and 6.5–7.0 min (5–5% B). The flow rate was 0.3 mL/min. 5 μL prepared sample was injected to the UPLC-MS/MS system. The autosampler tray and the column were maintained at 8°C and 35°C, respectively.

**FIGURE 2:**
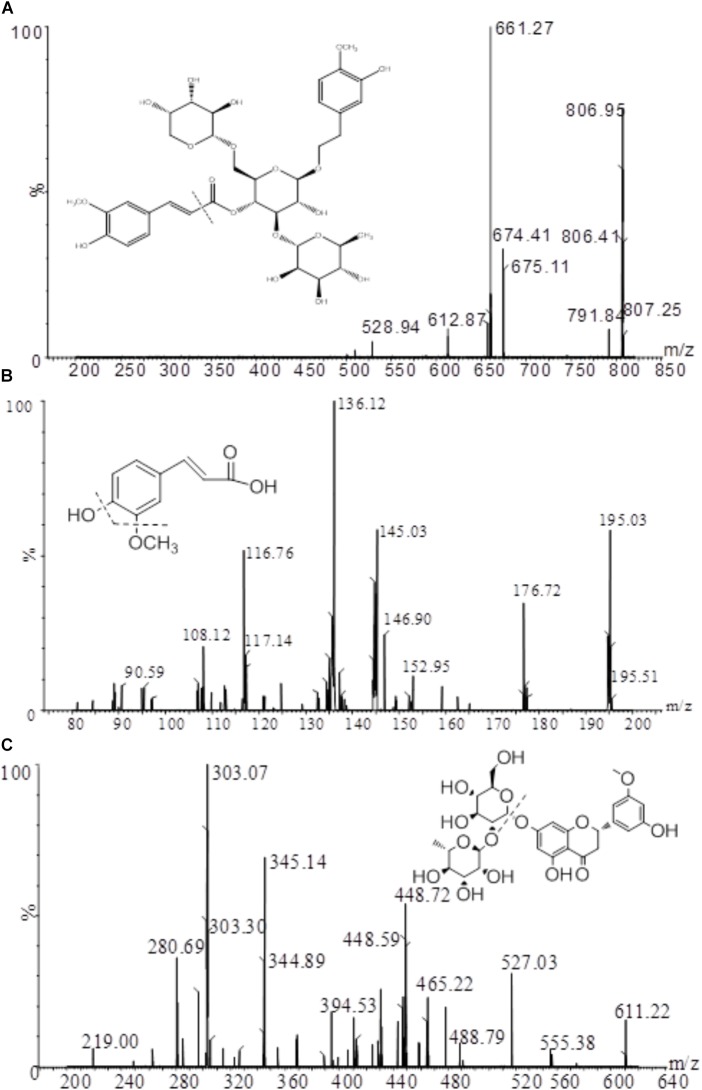
Mass fragmentation pattern of angoroside C **(A)**, ferulic acid **(B)**, and IS **(C)**.

A XEVO TQ triple quadrupole mass spectrometer equipped with an electro-spray ionization (ESI) source (Waters Corp., Milford, MA, United States) was used for mass spectrometric detection. Quantitation was achieved by UPLC-MS/MS detection in the positive ion mode for analytes and IS using the multiple reactions monitoring (MRM) mode under unit mass resolution in the mass analyzer. The optimal MS parameters were set as follows: capillary voltage 0.5 kV; desolvation temperature 400°C; source temperature 150°C; desolvation gas flow rate 800 L/h. The specific parameters for each analyte and IS are displayed in Table 1. The fragmentation pattern for angoroside C, ferulic acid and the IS in positive ion mode is shown in Figure 2. Masslynx 4.1 software (Waters Corp., Milford, MA, United States) was used for data acquisition and instrument control.

**Table 1:** Optimized mass parameters for angoroside C, ferulic acid, and the IS.

Analytes	Parent (m/z)	Daughter (m/z)	Dwell (s)	Cone (V)	Collision (V)
Angoroside C	807.25	661.27^a^	0.025	78	46
	807.25	529.19^b^	0.025	78	50
Ferulic acid	195.03	145.03^a^	0.025	18	16
	195.03	117.05^b^	0.025	18	22
Neohesperidin (IS)	611.22	303.07^a^	0.025	14	20

*^a^Ion for quantification and ^b^ion for qualitation.*

### Calibration Standards (CS) and Quality Control (QC) Sample Preparation

Stock solutions of angoroside C and ferulic acid (1,000 μg/mL) for CS and QC samples were prepared separately in 50% acetonitrile (containing 0.1% formic acid) by accurately weighing the working standard. These were then appropriately diluted in 50% acetonitrile (containing 0.1% formic acid) to prepare working standards for angoroside C and ferulic acid in the range from 4.88 to 10,000 ng/mL and 1.22 to 2,500 ng/mL, respectively. Then, 20 μL of each working standard solution was spiked into 100 μL of blank plasma, and the mixture was processed as described under the section of sample preparation, finally, we achieved twelve different concentrations for the plasma CS: 0.98, 1.95, 3.91, 7.81, 15.63, 31.25, 62.5, 125, 250, 500, 1,000, and 2,000 ng/mL for angoroside C, and 0.24, 0.49, 0.98, 1.95, 3.91, 7.81, 15.63, 31.25, 62.5, 125, 250, and 500 ng/mL for ferulic acid, The same procedure was followed to prepare three plasma QC samples at 2.5, 75, and 1,500 ng/mL for angoroside C, and 0.6, 20, and 400 ng/mL for ferulic acid, which were treated as the low, middle, and high QC samples (LQC, MQC, and HQC), respectively. A stock solution of IS (1,000 μg/mL) was also prepared and diluted in 50% acetonitrile (containing 0.1% formic acid) to obtain a working solution of 1,000 ng/mL. All stock and aqueous solutions were stored in a refrigerator (4°C), whereas spiked plasma samples were maintained at -80°C.

### Sample Preparation

A simple protein precipitation method was applied to extract angoroside C and ferulic acid from rat plasma and tissue homogenate. Briefly, the plasma or tissue homogenates were thawed to room temperature, and then 20 μL of IS solution and 20 μL of solvent (the volume of the corresponding working solution for calibration curve and QC samples) were added to 100 μL of the plasma sample or tissue homogenate sample (all weighed tissue sample was homogenized with ice-cold saline solution (1:3, w/v). The mixture was then vortexed for 3 min and extracted with 400 μL acetonitrile by vortex-mixing for 5 min. The upper layer was transferred to a clean tube after centrifugation at 14,000 rpm for 10 min. The organic phase was evaporated to dryness under a gentle stream of nitrogen gas. The obtained residue was reconstituted in 100 μL 50% acetonitrile–distilled water (containing 0.1% formic acid) and centrifuged at 14,000 rpm for another 10 min. The above operations were carried out at room temperature. Then, 5 μL aliquots were injected into the UPLC-MS/MS system for analysis.

### Animal Study

Sprague-Dawley (SD) rats (male, 200 ± 10 g, 8 weeks old) were obtained from the Hubei University of Medicine Animal Laboratory (Shiyan, China) and kept in an environmentally controlled room (temperature: 25 ± 2°C, humidity: 50 ± 5%, 12 h dark–light cycle) for at least 1 week before the experiments. The rats were fasted overnight before the day of the experiment. The animal protocols used in this study were approved by the Hubei University of Medicine’s Institutional Animal Care and Uses Committee.

For the pharmacokinetic study, oral dosing solutions were prepared in physiological saline at a concentration of 50 mg/mL. Intravenous dosing solution was prepared in physiological saline at a concentration of 10 mg/mL. Ten rats were randomly assigned to two groups (five rats/group) for the intravenous administration of 5 mg/kg angoroside C and the oral administration of 100 mg/kg angoroside C, respectively. Before blood sampling, animal were slightly anesthetized with inhalation of diethyl ether. Blood samples, approximately 150 μL each, were collected from the suborbital vein into heparinised tubes at 0, 5, 15, and 30 min, and at 1, 2, (or 3), 4, 6, 8, 12, and 24 h after dosing, and then centrifuged at 14,000 rpm for 10 min. All of the samples were stored at -80°C.

For the tissue distribution study, 20 male rats received a single oral administration of angoroside C at 100 mg/kg. According to the United States Food and Drug Administration (FDA) guidelines, at least three time points should be selected to represent the drug distribution of the absorption phase, the equilibrium phase, and the elimination phase, respectively, and referring to the trend of the plasma concentration-time curve of the pharmacokinetic study. Consequently, the tested animals were sacrificed by bleeding the femoral artery at 0.17, 0.50, 1.5, and 6 h (five rats at each time point), and the tissues including the heart, liver, spleen, lung, kidney, and brain were immediately excised and blood samples were collected simultaneously. All of the tissues were washed with cold physiological saline (4°C), excess fluid was blotted, and samples were accurately weighed and subjected to the processing described in the sample preparation section.

The pharmacokinetic parameters of angoroside C and the metabolite in plasma were calculated using non-compartmental analysis with DAS (Drug and Statistics) version 3.2.7 software (edited by the Chinese Mathematical Pharmacology Society). The maximum concentration (*C*_max_) and time to *C*_max_ (*T*_max_) was obtained from the concentration time plot, etc. The absolute oral bioavailability (F) of the analytes are measured by comparing each AUC_0-∞_ value after i.g. and i.v. administration according to the equation: F = (AUC_i.g._/Dose_i.g._)/(AUC_i.v._/Dose_i.v._).

### Method Validation

The method was validated in terms of specificity, calibration, extraction recovery, matrix effect, accuracy, intra-day, and inter-day precisions and stability, according to the united states FDA bioanalytical method validation guidance.

#### Specificity

The specificity of the method was evaluated by analysing six batches of blank rat plasma or liver homogenate, plasma samples or liver homogenate spiked with angoroside C and ferulic acid (LLOQ) and IS, and plasma samples or liver homogenate after oral administration to exclude potential endogenous interference.

#### Linearity of Calibration Curve and LLOQ

Linearity was determined by plotting the peak area ratio (*y*) of analytes to IS against the concentrations of the calibration standards (*x*) with weighted (1/*x*^2^) least square linear regression. The LLOQ was defined as the lowest concentration on the calibration curve that could fulfill the analytical requirement of S/N > 10 with an acceptable accuracy and precision (within ±20%).

#### Recovery and Matrix Effect

Recovery of analytes was evaluated by comparing the peak area from six replicates QC samples at low, medium, and high concentrations that were spiked with analytes prior to extraction with the peak area of those that were spiked with blank plasma or liver homogenate. The matrix effects were evaluated by comparing the peak areas of the analytes in post-extracted blank plasma samples spiked with QC samples, the pure standard solutions with same concentration were dried directly and reconstituted with the mobile phase.

#### Precision and Accuracy

Intra- and inter-day variations were carried out by analysing six replicate samples (six same samples were prepared simultaneously) for each QC level on three consecutive validation days. The precision and accuracy was expressed as the relative standard deviation (RSD%) and relative error (RE%), respectively.

#### Stability

The stability of analytes was evaluated by injecting three replicate samples for each QC level stored under different conditions. Short-term stability was assessed using samples kept at room temperature for 6 h. Long-term stability was determined by analysing the QC samples kept at the storage temperature (-80°C) for 2 weeks. The freeze-thaw stability was determined after three freeze-thaw cycles (-80°C to room temperature was considered one cycle), and the auto-sampler stability of the extracted samples was evaluated at 8°C for 8 h.

## Results and Discussion

### Method Development

#### Specificity

**FIGURE 3:**
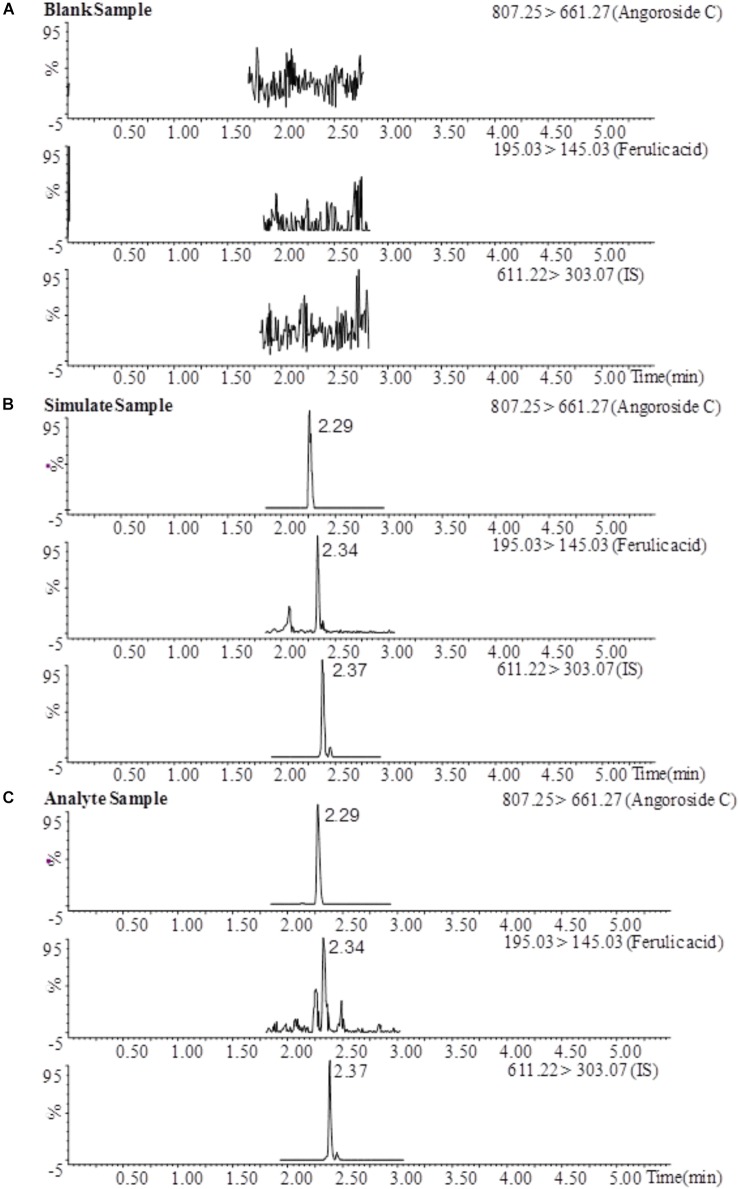
MRM chromatograms of angoroside C, ferulic acid, and IS in a blank plasma sample **(A)**, a blank plasma sample spiked with analytes (LLOQ) and IS **(B)**, and a plasma sample 1 h after oral administration of angoroside C **(C)**.

The typical MRM chromatograms of angoroside C and ferulic acid in blank plasma, blank plasma spiked with standards and rat plasma samples after the oral administration of angoroside C are shown in Figure 3. Angoroside C, ferulic acid and IS were detected at 2.29 min, 2.34 and 2.37 min, respectively. No significant interference with the test compound was observed with the endogenous material in the rat plasma, and similar phenomena have also been observed in tissue homogenates. Therefore, the specificity was sufficient to accurately characterize the pharmacokinetics and tissue distribution of angoroside C and ferulic acid.

#### Linearity and Calibration Curve

Regression equations and correlation of coefficients for angoroside C and ferulic acid in different tissue homogenate and plasma are listed in Table 2. The calibration curves of the two analytes were linear with correlation coefficients (*r*) > 0.99, and the linear ranges were 0.98–2,000 ng/mL and 0.24–500 ng/mL for angoroside C and ferulic acid, respectively. The LLOQs for angoroside C and ferulic acid were 0.98 and 0.24 ng/mL, respectively.

**Table 2:** Regression equations of angoroside C and ferulic acid in different samples.

Tissues	Angoroside C		Ferulic acid	
	Standard curve	*R*^2^	Standard curve	*R*^2^
Plasma	*y* = 0.0196*x* + 0.0038	0.9988	*y* = 0.0332*x* + 0.1167	0.9927
Heart	*y* = 0.008*x* - 0.0032	0.9929	**-**	**-**
Liver	*y* = 0.0037*x* + 0.001	0.9993	*y* = 0.0245*x* + 0.038	0.9992
Spleen	*y* = 0.007*x* - 0.0015	0.9964	*y* = 0.0313*x* + 0.0199	0.9973
Lung	*y* = 0.0092*x* - 0.0083	0.9981	*y* = 0.0313*x* + 0.0335	0.9975
Kidney	*y* = 0.0035*x* - 0.0034	0.9914	*y* = 0.0239*x* + 1.534	0.9918
Brain	*y* = 0.0088*x* + 0.0007	0.9962	**-**	**-**

#### Precision and Accuracy

The values of intra- and inter-day precision and accuracy for angoroside C and ferulic acid are shown in Table 3. The RSD and RE values were typically <15% for all analytes. These data demonstrate that the developed method was reliable and reproducible.

**Table 3:** Precision and accuracy of angoroside C and ferulic acid in rat plasma and liver homogenate.

Samples	Analytes	Spiked conc. (ng/mL)	Precision (RSD %)		Accuracy (%)
			Intra-day	Inter-day	
Plasma	Angoroside C	2.5	14.6	13.5	86.5 14.7
		75	13.3	3.9	79.8 10.5
		1,500	9.9	5.8	81.4 13.2
	Ferulic acid	0.6	14.9	12.7	91.3 9.6
		20	13.2	12.8	84.2 13.9
		400	6.2	9.4	103.5 7.3
Liver	Angoroside C	2.5	8.5	5.5	113.2 9.4
		75	10.8	7.4	108.1 14.9
		1,500	5.2	10.6	75.2 8.7
	Ferulic acid	0.6	13.7	11.8	113.4 12.5
		20	11.1	9.2	106 5.8
		400	4.7	5.9	101.9 8.3

*Values are means ± SD (n = 5).*

#### Recovery and Matrix Effect

The extraction recoveries and matrix effects of angoroside C and ferulic acid are shown in Table 4. The extraction recoveries of each analyte at different concentrations were consistent and no significant matrix effect was detected in rat plasma and liver homogenate.

**Table 4:** Matrix effects and extract recoveries of angoroside C and ferulic acid in rat plasma and liver homogenate (*n* = 5).

Samples	Analytes	Spiked conc. (ng/mL)	Recovery		Matrix effect	
			Mean (%)	RSD (%)	Mean (%)	RSD (%)
Plasma	Angoroside C	2.5	82.7	11.4	95.4	8.4
		75	87.4	11.5	112.7	12.8
		1,500	100.0	14.9	100.7	6.8
	Ferulic acid	0.6	87.4	12.8	97.6	5.8
		20	74.8	13.2	107.3	5.7
		400	79.2	7.5	94.3	9.2
Liver	Angoroside C	2.5	103.5	12.6	112.3	10.5
		75	94.7	9.3	96.5	6.6
		1,500	113.2	5.5	80.7	8.7
	Ferulic acid	0.6	79.5	5.8	107.4	9.7
		20	83.4	10.1	86.3	13.8
		400	78.9	9.3	85.2	14.4

#### Stability

The results of the stability experiments are summarized in Table 5. Angoroside C and ferulic acid were stable in plasma samples and liver homogenate stored at -80°C for 14 days and after three freeze-thaw cycles. Additionally, the processed plasma samples were stable at room temperature for 6 h, and the extracted samples were stable in an auto-sampler at 8°C for 8 h. The RSD values were <15% for all analytes.

**Table 5:** Stability (RE, %) of angoroside C and ferulic acid in rat plasma and liver homogenate at different storage conditions (*n* = 5).

Samples	Analyte	Spiked conc. (ng/mL)	3 freeze-thaw cycles	6 h at room temperature	8 h in the autosampler	-80°C for 2 weeks
Plasma	Angoroside C	2.5	3.5	-4.6	13.7	7.6
		75	-9.2	7.9	4.8	9.3
		1,500	5.4	5.3	6.4	-2.5
	Ferulic acid	0.6	7.3	12.8	-3.1	1.7
		20	-12.3	14.6	-5.8	13.2
		400	6.8	4.7	-14.5	8.8
Liver	Angoroside C	2.5	7.4	-6.4	5.4	5.7
		75	-9.5	2.6	1.3	3.4
		1,500	11.4	11.7	5.2	-8.2
	Ferulic acid	0.6	12.5	9.9	7.4	-12.4
		20	8.5	6.5	2.8	-14.1
		400	-3.3	-8.4	9.1	9.9

### Pharmacokinetics Study

**FIGURE 4:**
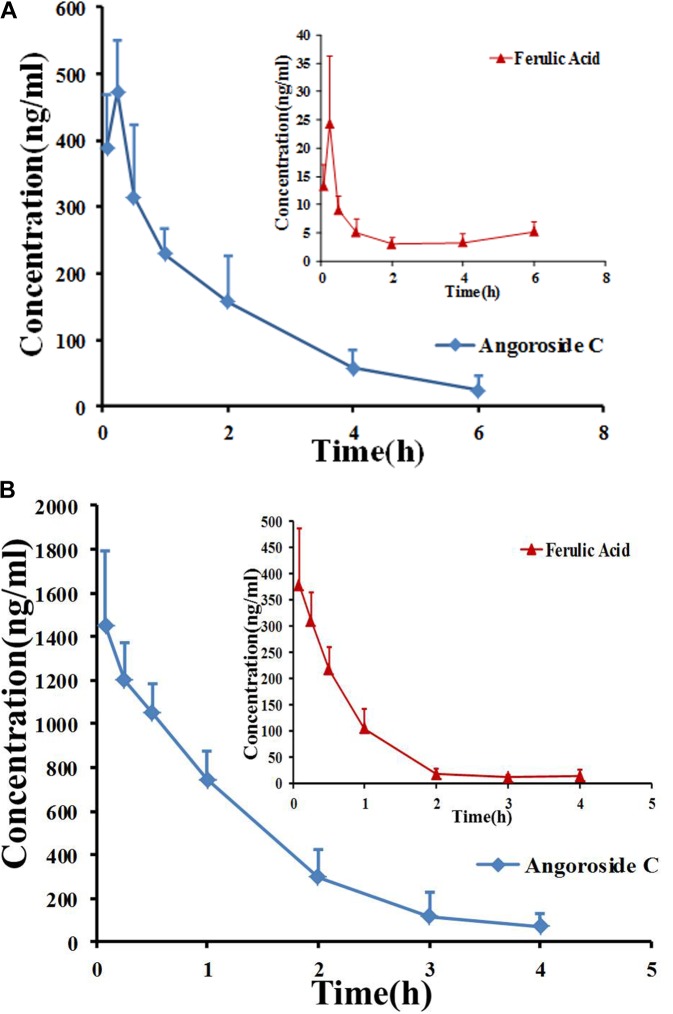
Mean plasma concentration–time curves for angoroside C and ferulic acid in rats after intragastric administration of angoroside C at 100 mg/kg **(A)** and intravenous administration at 5 mg/kg **(B)**. Values are means ± SD (*n* = 5).

The developed and validated UPLC-MS/MS method was successfully applied for the determination of angoroside C and ferulic acid pharmacokinetics and tissue distribution. After the intravenous injection of 5 mg/kg angoroside C or the oral administration of 100 mg/kg angoroside C to rats, angoroside C and its metabolite ferulic acid were detected in plasma samples. The plasma concentration-time profiles of the two analytes are presented in Figures 4A,B, and the main pharmacokinetic parameters are listed in Table 6. As shown in Figure 4A and Table 6, after the oral administration of angoroside C, its plasma concentration increased sharply, and reached a peak concentration at approximately 15 min with the *C*_max_ of 473.5 ± 77.6 ng/mL, which indicated that angoroside C was rapidly absorbed, and rapidly eliminated with the *t*_1/2z_ of 1.26 ± 0.18 h. The AUC values for the oral and intravenous administration of angoroside C were 812.0 ± 216.1 and 1,901.3 ± 469.9 min mg/mL, respectively. The calculated absolute oral bioavailability (F) of angoroside C in rats was 2.1%. The *C*_max_ of ferulic acid was only 24.38 ± 11.9 ng/mL at 15 min after the oral administration of 100 mg/kg angoroside C. However, the highest concentration of ferulic acid in the collected plasma sample was 382.57 ± 108.9 ng/mL after the intravenous administration of 5 mg/kg angoroside C. Thus, angoroside C may be metabolized to ferulic acid by carboxylesterase in rat plasma. The *AUC_0-∞_* (Figure 4B) of angoroside C (2,000.6 ± 567.6 mg⋅min/L) was about 6.4-fold that of ferulic acid (313.72 ± 70.94 mg⋅min/L), suggesting that ferulic acid may be the main metabolite of angoroside C in plasma.

**Table 6:** Pharmacokinetic parameters of angoroside C and ferulic acid in rat plasma after oral administration of angoroside C at 100 mg/kg and intravenous administration at 5 mg/kg.

Parameters	Intravenous		Oral	
	Angoroside C	Ferulic acid	Angoroside C	Ferulic acid
AUC_(0-t)_ (ng h/mL)	1,901.3 ± 469.9	299.19 ± 68.1	812.0 ± 216.1	15.56 ± 3.61
AUC_(0-∞)_ (ng h/mL)	2,000.6 ± 567.6	313.72 ± 70.94	842.4 ± 230.6	19.34 ± 5.04
MRT_(0-t)_ (h)	0.99 ± 0.16	0.53 ± 0.03	1.61 ± 0.27	0.65 ± 0.05
MRT_(0-∞)_ (h)	1.17 ± 0.33	0.63 ± 0.04	1.82 ± 0.37	1.17 ± 0.48
*t*_1/2z_ (h)	0.82 ± 0.29	0.46 ± 0.13	1.26 ± 0.18	0.83 ± 0.39
*T*_max_ (h)	0.0833 ± 0.0000	0.13 ± 0.08	0.25 ± 0.00	0.25 ± 0.00
*C*_max_ (ng/mL)	1,452.8 ± 343.1	382.57 ± 108.9	473.5 ± 77.6	24.38 ± 11.9
Bioavailability (%)			2.1%	

*Values are means ± SD (n = 5).*

### Tissue Distribution Study

**FIGURE 5:**
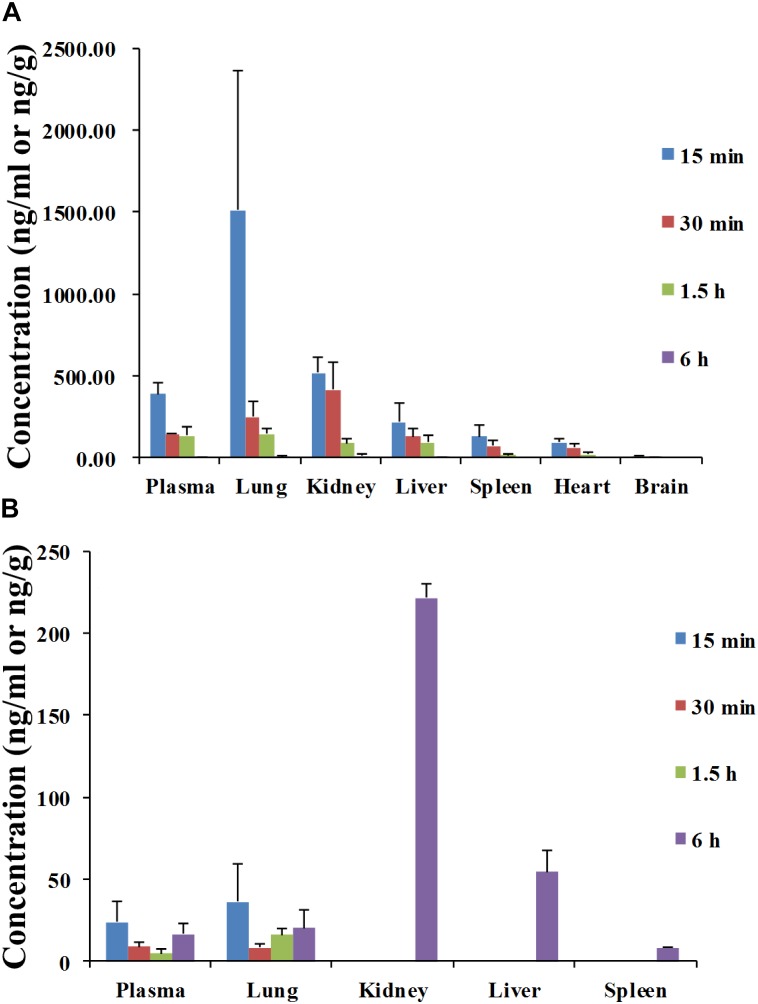
Mean concentration–time profiles of angoroside C **(A)** and ferulic acid **(B)** in plasma (ng/mL), liver, heart, spleen, lung, kidney, and brain tissues (ng/g) after intragastric administration of angoroside C in different time at a dose of 100 mg/kg to rat. Values are means ± SD (*n* = 5).

The tissue distributions of angoroside C and ferulic acid were investigated in male rats at 0.25, 0.5, 1.5, and 6 h after oral administration at a dose of 100 mg/kg of angoroside C. As shown in Figure 5A, angoroside C is widely distributed in all of the main organs, including the liver, heart, spleen, lung, kidney, and brain, with the highest concentrations of angoroside C detected in the lung at 15 min after administration, followed by the kidney, liver, spleen, and brain. Similar to its plasma pharmacokinetics, angoroside C is distributed rapidly and cleared from all of the organs at almost 6 h. We also detected ferulic acid in various tissues (Figure 5B), with the highest concentrations in different tissues observed at 6 h after dosing in rats; there maybe some metabolic conversion in the rat intestine or liver. Additionally, in our current study, no ferulic acid was found in the brain, which was not complete coincident with the previous report (Gasperotti et al., 2015). This discrepancy may be due to the differences in dosage of use, route of administration or blood–brain barrier permeability change induced by multi-component mixture simultaneous administration. Nonetheless, the nephrotropic behavior of ferulic acid is in agreements with the previous study noted above.

## Conclusion

In our study, a fast, simple and sensitive UPLC-MS/MS method for the separation and simultaneous determination of angoroside C and its metabolite ferulic acid in rat plasma and tissue homogenate was developed and validated for the first time. Angoroside C and ferulic acid were rapidly analyzed within 4 min. The short run time and simple pre-treatment procedure made it easy and fast to perform. The method was then successfully applied to assess the pharmacokinetics, bioavailability and tissue distribution of angoroside C and ferulic acid in rats after administration by different routes. The studies have shown that angoroside C could be metabolized to ferulic acid in the body. Meanwhile, some potential metabolites of angoroside C, such as hydroxytyrosol, 3-hydroxyphenylpropionic acid, 3-phenylpropionic acid, 3,4-dihydroxybenzenepropionic acid, and homovanillic acid (Pasdaran and Hamedi, 2017) also be investigated in this experiment, nevertheless, none of them were detected in rat plasma or tissues.

According to the analysis of the chemical structure of angoroside C, there are several phenolic hydroxyl groups, ester bonds and glycosidic bonds, which mean that it can be easily oxidized and degraded. According to some studies of similar structural compounds such as echinacoside and acteoside, phenylethanoid glycosides exhibit extensive strong activity. However, clear pharmacological characteristics such as poor permeability, fast and extensive metabolism in the gut, liver, or blood, indicate that PhGs may be potential pro-drugs for treating several diseases. The exact contribution of action of the parent and metabolites are still elusive and require special attention in the future.

## Author Contributions

LC and TZ conceived and designed the study. CZ, LC, and TZ analyzed the research data and wrote the manuscript. CZ, WM, YZ, QW, CQ, SD, LH, FY, and TZ carried out the experiments.

## Conflict of Interest Statement

The authors declare that the research was conducted in the absence of any commercial or financial relationships that could be construed as a potential conflict of interest.

## References

[B1] Cano E. Veiga M. Jiménez C. Riguera R. (1990). Pharmacological effects of three phenylpropanoid glycosides from *Mussatia*. *Planta Med.* 56 24–26. 10.1055/s-2006-960876 2356239

[B2] Díaz A. M. Abad M. J. Fernández L. Silván A. M. De Santos J. Bermejo P. (2004). Phenylpropanoid glycosides from *Scrophularia scorodonia*: in vitro anti-inflammatory activity. *Life Sci.* 74 2515–2526. 10.1016/j.lfs.2003.10.008 15010262

[B3] Gasperotti M. Passamonti S. Tramer F. Masuero D. Guella G. Mattivi F. (2015). Fate of microbial metabolites of dietary polyphenols in rats: is the brain their target destination? *ACS Chem. Neurosci.* 8 1341–1352. 10.1021/acschemneuro.5b00051 25891864

[B4] Gu W. L. Chen C. X. Huang X. Y. Gao J. P. (2015). The effect of angoroside C on pressure overload-induced ventricular remodeling in rats. *Phytomedicine* 22 705–712. 10.1016/j.phymed.2015.05.002 26141756

[B5] Gu W. L. Chen C. X. Wu Q. Lü J. Liu Y. Zhang S. J. (2010). Effects of Chinese herb medicine radix scrophulariae on ventricular remodeling. *Pharmazie* 65 770–775. 10.1691/ph.2010.0561 21105581

[B6] Huang C. G. Li Y. M. He X. (2004). Effect of phenylpropanolid glycosides of *Scrophularia ningpoensis* on hepatocellular apoptosis in rats with acute liver injury. *Chin. J. Integr. Tradit. West Med. Live Dis.* 14 160–161. 10.3969/j.issn.1005-0264.2004.03.012

[B7] Huang X. Y. Chen C. X. Zhang X. M. Liu Y. Wu X. M. Li Y. M. (2012). Effects of ethanolic extract from radix scrophulariae on ventricular remodeling in rats. *Phytomedicine* 19 193–205. 10.1016/j.phymed.2011.09.07 22035768

[B8] Liu H. Zheng Y. F. Li C. Y. Zheng Y. Y. Wang D. Q. Wu Z. (2015). Discovery of anti-inflammatory ingredients in Chinese herbal formula kouyanqing granule based on relevance analysis between chemical characters and biological effects. *Sci. Rep.* 5:18080. 10.1038/srep18080 26657159 PMC4674803

[B9] Liu X. Li X. Ji S. Cui X. Li M. (2016). Screening of bioactive ingredients in ligusticum chuanxiong hort for protection against myocardial ischemia. *Cell. Physiol. Biochem.* 40 770–780. 10.1159/000453137 27915331

[B10] Liu Y. Chi S. Wang W. Su L. Liu B. (2017a). Simultaneous determination of seven components in rat plasma by the uplc-ms/ms method and application of pharmacokinetic studies to simiaoyong′an decoction. *Molecules* 22:E1937. 10.3390/molecules22111937 29120359 PMC6150365

[B11] Liu Y. M. Hu C. Y. Shen J. D. Wu S. H. Li Y. C. Yi L. T. (2017b). Elevation of synaptic protein is associated with the antidepressant-like effects of ferulic acid in a chronic model of depression. *Physiol. Behav.* 169 184–188. 10.1016/j.physbeh.2016.12.003 27940143

[B12] Li Y. M. Jiang S. H. Gao W. Y. Zhu D. Y. (2000). Phenylpropanoid glycosides from *Scrophularia ningpoensis*. *Phytochemistry* 54 923–925. 10.1016/S0031-9422(00)81758-6 11014290

[B13] Morikawa T. Pan Y. Ninomiya K. Imura K. Matsuda H. Yoshikawa M. (2010). Acylated phenylethanoid oligoglycosides with hepatoprotective activity from the desert plant *Cistanche tubulosa*. *Bioorg. Med. Chem.* 18 1882–1890. 10.1016/j.bmc.2010.01.047 20159656

[B14] National Pharmacopoeia Committee (2015). *Pharmacopoeia of the People’s Republic of China, Part1.* Beijing: Chemical Industry Press.

[B15] Panda V. Laddha A. Nandave M. Srinath S. (2016). Dietary phenolic acids of *Macrotyloma uniflorum* (horse gram) protect the rat heart against isoproterenol-induced myocardial infarction. *Phytother. Res.* 30 1146–1155. 10.1002/ptr.5620 27091200

[B16] Pasdaran A. Hamedi A. (2017). The genus *Scrophularia*: a source of iridoids and terpenoids with a diverse biological activity. *Pharm. Biol.* 55 2211–2233. 10.1080/13880209.2017.1397178 29125010 PMC6130519

[B17] Perezternero C. Werner C. M. Nickel A. G. Herrera M. D. Motilva M. J. Böhm M. (2017). Ferulic acid, a bioactive component of rice bran, improves oxidative stress and mitochondrial biogenesis and dynamics in mice and in human mononuclear cells. *J. Nutr. Biochem.* 48 51–61. 10.1016/j.jnutbio.2017.06.011 28759787

[B18] Qian J. Hunkler D. Safayhi H. Rimpler H. (1991). New iridoid-related constituents and the anti-inflammatory activity of *Scrophularia ningpoensis*. *Planta Med.* 57:A56 10.1055/s-2006-960316

[B19] Salazar-López N. J. Astiazarán-García H. González-Aguilar G. A. Loarca-Piña G. Ezquerra-Brauer J. M. Avila J. A. D. (2017). Ferulic acid on glucose dysregulation, dyslipidemia, and inflammation in diet-induced obese rats: an integrated study. *Nutrients* 9:e675. 10.3390/nu9070675 28661434 PMC5537790

[B20] Tian Y. S. Du Z. Y. Xiao Y. Yu B. Qi J. (2017). Screening and identification of potential hypoglycemic components in Zeng Ye Tang by high-performance liquid chromatography coupled with tandem quadrupole time-of-flight mass spectrometry. *J. Sep. Sci.* 40 4709–4717. 10.1002/jssc.201700507 29098768

[B21] Wagner H. Bauer R. Merlchart D. Xiao P. G. Staudinger A. (2011). *Chromatographic Fingerprint Analysis of Herbal Medicines (TLC and HPLC of Chinese Drugs). Vol. II.* New York, NY: Springer Wien 10.1007/978-3-7091-0763-8

[B22] Xu F. Q. Xu X. D. Chen S. L. (2013). Research progress on the chemical components and pharmacological activities of *Scrophularia ningpoensis* Hemsl.. *Modern Chin. Med.* 15 752–759. 10.13313/j.issn.1673-4890.2013.09.020

[B23] Yang X. Yang S. Zhang X. Li L. (2009). Determination of five compounds in *Scrophularia ningpoensis* by HPLC-UV-ELSD. *China J. Chin. Mater. Med.* 34 68–71. 19382455

[B24] Zhang Q. Lei H. M. Wang P. L. Ma Z. Q. Zhang Y. Wu J. J. (2017). Bioactive Components from qingwen baidu decoction against LPS-induced acute lung injury in rats. *Molecules* 22:e692. 10.3390/molecules22050692 28445422 PMC6154387

[B25] Zhang X. Wang R. An R. Wu X. Wang X. Li Y. (2011). Simultaneous determination of five constituents in *Scrophularia ningpoensis* by HPLC. *China J. Chin. Mater. Med.* 36 709–711. 21710734

